# Complete Genome Sequences of 37 Bacteria from the Human Vaginal Tract

**DOI:** 10.1128/mra.00358-23

**Published:** 2023-05-23

**Authors:** Christopher Whidbey, Alyssa N. Konopaski, Robel Teshome, Sejal Dhaliwal, M. Quinn Peters, Marlyd E. Mejia, Mallory B. Ballard, Kathryn A. Patras

**Affiliations:** a Department of Chemistry, Seattle University, Seattle, Washington, USA; b Department of Molecular Virology and Microbiology, Baylor College of Medicine, Houston, Texas, USA; c Alkek Center for Metagenomics and Microbiome Research, Baylor College of Medicine, Houston, Texas, USA; University of Rochester School of Medicine and Dentistry

## Abstract

Microorganisms colonizing the human vaginal mucosa are associated with healthy states, as well as conditions such as bacterial vaginosis and infection-associated preterm birth. Here, we report complete genome sequences of 37 bacterial isolates from the human vaginal tract.

## ANNOUNCEMENT

The vaginal microbiota is typically characterized by low α-diversity and dominance by a single *Lactobacillus* sp. (1, 2). However, individuals with diverse vaginal communities of facultative and obligate anaerobes are at increased risk for adverse health outcomes (3–5). While vaginal communities correlate with host health status, the genetic basis for this correlation is poorly understood. To better understand the content and structure of vaginal bacterial genomes, we sequenced 37 human vaginal isolates using the Oxford Nanopore Technologies (ONT) platform.

Isolates were obtained with informed consent from midvaginal swabs from healthy human donors (*n* = 22) between 23 and 64 years of age (Baylor College of Medicine Institutional Review Board [IRB] number H-47537). Donors included Black (*n* = 2), Asian (*n* = 5), Hispanic/Latino (*n* = 3), and white women (*n* = 12). Swabs were suspended in prereduced phosphate buffered saline, and the suspension was plated onto de Man, Rogosa, and Sharpe agar or tryptic soy agar with 5% sheep’s blood, as indicated (Table 1). After 48 to 72 h of microoxic (5% O_2_) or anaerobic (<5 ppm O_2_) incubation at 37°C, the strains were isolated and lyophilized. No strain identification was performed prior to genome sequencing.

**TABLE 1:** Strain information and genome metrics for vaginal bacterial isolates

Sample name	Species	SRA accession no.	Assembly data			GenBank accession no.	No. of genes	Coverage (×)	GC content (%)	No. of replicons	Chromosome data		Plasmid data		Culture conditions	Batch no.
			No. of reads	*N*_50_ (bp)	Size (bp)						Size (bp)	No. of CDSs^*a*^	Size(s) (bp)	No. of CDSs		
VSI01	Winkia neuii	SRX19553237	42,775	16,913	2,280,458	CP118948	2,085	184.126	56.67	1	2,280,458	2,022			MRS, anaerobic	1
VSI03	Aerococcus christensenii	SRX19553238	198,899	5,200	1,713,899	CP118095	1,646	447.154	39	1	1,713,899	1,570			MRS, anaerobic	1
VSI04	Lactobacillus crispatus	SRX19553249	180,062	8,541	2,340,820	CP118947	2,349	336.278	37.33	1	2,322,225	2,257			MRS, anaerobic	1
VSI05	Limosilactobacillus fermentum	SRX19553260	251,234	6,998	2,215,744	CP118092	2,259	504.253	50.98	1	2,215,744	2,183			MRS, anaerobic	1
VSI06	Lactobacillus gasseri	SRX19553268	169,209	5,668	1,819,995	CP118090, CP118091	1,722	366.215	35.09	2	1,776,923	1,628	43,072	51	MRS, anaerobic	1
VSI07	Lactobacillus gasseri	SRX19553269	94,548	8,941	1,815,045	CP118088, CP118089	1,718	289.026	35.1	2	1,771,973	1,624	43,072	52	MRS, anaerobic	1
VSI08	Lactobacillus crispatus	SRX19553270	163,850	9,365	2,342,983	CP118087	2,353	344.996	37.21	1	2,342,983	2,265			MRS, anaerobic	1
VSI09	Enterococcus faecalis	SRX19553271	22,723	21,127	3,041,350	CP118085, CP118086	2,933	88.9462	37.45	2	2,984,075	2,857	57,275	68	MRS, anaerobic	1
VSI10	*Winkia neuii*	SRX19553272	18,673	20,754	2,217,156	CP118946	2,039	95.583	56.65	1	2,217,156	1,976			MRS, anaerobic	1
VSI11	Bifidobacterium breve	SRX19553273	32,826	18,183	2,536,081	CP118083	2,358	122.511	58.85	1	2,536,081	2,285			MRS, anaerobic	1
VSI12	Streptococcus anginosus	SRX19553239	22,848	15,695	1,926,669	CP118082	1,958	126.798	38.8	1	1,926,669	1,884			MRS, anaerobic	1
VSI13	Streptococcus anginosus	SRX19553240	44,455	19,420	1,926,555	CP118081	1,955	241.067	38.8	1	1,926,555	1,881			MRS, anaerobic	1
VSI14	Streptococcus agalactiae	SRX19553241	37,528	10,770	2,201,661	CP118080	2,258	139.765	35.72	1	2,201,661	2,154			MRS, anaerobic	1
VSI15	Streptococcus agalactiae	SRX19553242	39,966	8,482	2,201,662	CP118079	2,253	112.687	35.72	1	2,201,662	2,149			MRS, anaerobic	1
VSI16	Streptococcus anginosus	SRX19553243	47,592	19,839	2,085,682	CP118078	2,126	229.199	38.66	1	2,085,682	2,052			MRS, anaerobic	1
VSI17	Lactobacillus crispatus	SRX19553244	131,686	5,996	2,455,419	CP118077	2,459	184.735	37.14	1	2,455,419	2,459			MRS, anaerobic	1
VSI18	Lactobacillus mulieris	SRX19553245	71,151	9,332	1,813,263	CP118076	1,814	208.443	34.45	1	1,813,263	1,744			MRS, anaerobic	1
VSI19	Enterococcus faecalis	SRX19553246	168,952	8,193	2,928,933	CP118072-5	2,731	252.572	37.61	4	2,795,902	2,654	70,631; 62,400; 59,239	75, 67, 66	MRS, anaerobic	1
VSI20	Bifidobacterium bifidum	SRX19553247	38,996	14,337	2,238,598	CP118071	1,901	79.2565	62.76	2	2,238,598	1,838			MRS, anaerobic	1
VSI21	Lactobacillus crispatus	SRX19553248	128,001	5,884	2,476,997	CP118069, CP118070	2,520	162.811	37.26	2	2,460,334	2,433	16,663	21	MRS, anaerobic	1
VSI24	Lactobacillus crispatus	SRX19553250	49,894	7,851	2,269,013	CP118067, CP118068	2,271	95.9306	37.21	2	2,252,356	2,189	16,657	20	MRS, anaerobic	1
VSI26	Lactobacillus gasseri	SRX19553251	49,732	3,280	1,948,711	CP118065, CP118066	1,864	67.0232	35.28	2	1,892,613	1,780	56,098	46	MRS, anaerobic	1
VSI33	Lacticaseibacillus rhamnosus	SRX19553265	9,717	14,305	2,991,185	CP118035	2,772	29.4481	46.76	1	2,991,185	2,692			MRS, microoxic	2
VSI35	Staphylococcus haemolyticus	SRX19553252	11,838	20,131	2,453,132	CP118060-4	2,392	41.3371	32.85	5	2,406,705	2,308	21,804; 20,842; 4,316; 3,781	22, 23, 6, 5	Blood agar, microoxic	2
VSI36	Enterococcus faecalis	SRX19553253	72,503	2,888	3,006,678	CP118057-9	2,846	52.2164	37.54	3	2,940,442	2,769	61,093; 5,143	68, 5	Blood agar, microoxic	2
VSI37	Streptococcus anginosus	SRX19553254	41,092	7,017	2,155,669	CP118054-6	2,186	84.7119	39.03	3	2,138,327	2,112	12,985; 4,357	13, 4	MRS, microoxic	2
VSI40	Streptococcus anginosus	SRX19553255	24,453	2,047	1,958,159	CP118053	2,015	24.4568	39.03	1	1,958,159	1,941			Blood agar, microoxic	2
VSI43	*Lacticaseibacillus rhamnosus*	SRX19553256	7,193	19,758	3,049,996	CP118050-2	2,789	26.7415	46.79	3	3,006,772	2,711	39,974; 3,250	45, 3	Blood agar, microoxic	2
VSI47	Lactobacillus gasseri	SRX19553264	10,365	7,438	2,038,797	CP118032-4	1,903	21.6542	35.21	3	1,903,801	1,812	84,857; 50,139	89, 53	MRS, microoxic	2
VSI48	Enterococcus faecalis	SRX19553257	16,902	19,662	2,755,036	CP118048, CP118049	2,556	57.3349	37.63	2	2,699,936	2,482	55,100	57	Blood agar, microoxic	2
VSI52	Streptococcus anginosus	SRX19553258	31,593	17,185	2,097,304	CP118046, CP118047	2,126	144.372	38.58	2	2,081,644	2,051	15,660	18	MRS, microoxic	2
VSI56	Staphylococcus aureus	SRX19553259	27,119	5,964	2,716,118	CP118044, CP118045	2,647	42.0185	32.86	2	2,698,209	2,565	17,909	23	Blood agar, microoxic	2
VSI63	Streptococcus agalactiae	SRX19553261	29,162	13,686	2,090,147	CP118043	2,135	39.5813	35.58	1	2,090,147	2,030			Blood agar, anaerobic	2
VSI67	Streptococcus anginosus	SRX19553266	4,993	15,492	2,091,268	CP118031	2,176	19.1477	39.11	1	2,091,268	2,102			Blood agar, microoxic	2
VSI68	Streptococcus anginosus	SRX19553267	66,624	3,446	2,010,423	CP118029	2,073	94.4345	38.84	1	2,010,423	1,998			MRS, microoxic	2
VSI78	Klebsiella pneumoniae	SRX19553262	30,930	5,898	5,523,217	CP118038-42	5,314	24.3744	57.57	5	5,213,267	4,968	139,214; 134,868; 31,157; 4,711	140, 151, 48, 7	Blood agar, anaerobic	2
VSI85	Streptococcus anginosus	SRX19553263	20,892	15,981	2,130,825	CP118036, CP118037	2,179	40.4093	39.19	2	2,130,825	2,101	4,746	4	Blood agar, anaerobic	2

a CDSs, coding DNA sequences.

Genomic DNA was isolated from lyophilized cell pellets (Zymo Research Quick-DNA high-molecular-weight [HMW] MagBead kit) following the manufacturer’s recommendations, except that mutanolysin (Sigma-Aldrich; 250 U/mL) was added during lysis. DNA was sheared with a 26-gauge needle, and integrity was confirmed using agarose gel electrophoresis. No size selection was performed. DNA was barcoded and prepared for sequencing using ONT ligation sequencing kits (batch 1: SQK-LSK109; batch 2: SQK-LSK114). Sequencing was performed using a MinION instrument (batch 1: R9.4.1 flow cell; 3.73 × 10^6^ reads; *N*_50_, 8,250 bp; batch 2: R10.4.1; 4.95 × 10^6^ reads; *N*_50_, 3,080 bp).

Base calling and demultiplexing were performed using Guppy (batch 1: v4.4.0; batch 2: v6.1.3). Default parameters were used unless otherwise noted. Filtlong v0.2.1 (6) was used for quality control (QC), filtering out reads of <1 kbp and retaining the top 95% of reads. Due to the low number of reads, the VSI67 genome was assembled using Flye v2.9.1 (7). All other genomes were assembled using Trycycler (batch 1: v0.4.1; batch 2: v0.5.3) (8). Subsamples were generated, and assemblies were performed using Flye (batch 1: v2.8.3; batch 2: v2.9.1) (7), Miniasm v0.3 (9), and Raven v1.5.1 (10). The resulting contigs were clustered and manually inspected for consistency (e.g., removing duplicate plasmids). The contigs were aligned using MUSCLE v3.8 (11), reconciled, and circularized (in some instances, the parameters max_add_seq_percent or max_trim_seq_percent were increased to 15). The reads were partitioned into contigs and used to generate a consensus sequence for the final assembly. All assemblies were polished once using Medaka v1.4.3 (12). Errors in ONT data typically occur at homopolymer sites, leading to erroneous insertions and deletions, subsequent frameshift mutations, and resulting pseudogenes. Because the assemblies were generated solely from ONT data, they should be used with this limitation in mind.

The resulting 37 closed genomes belong to commensals and pathobionts previously associated with the vaginal microbiome. Information regarding the final assemblies is provided in Table 1.

### Data availability.

The genomes and accompanying plasmid sequences have been deposited at NCBI GenBank and in the Sequence Read Archive under BioProject accession number PRJNA934404. The individual strain accession numbers are listed in Table 1.
